# Supplementary material for: How Mentoring Networks Shape Early-Career Grant Success: Evidence from NIH K-awardees
Source: bioRxiv. 2026 Aug 13:2026.08.11.743320. Preprint. [Version 1] doi: 10.64898/2026.08.11.743320 (PMC13640723; doi:10.64898/2026.08.11.743320)
Supplement: Supplementary file 1 [file media-1.pdf]

Table S1. Average proportion of mentors with a specific primary role being attributed to each type of mentoring outcomes. Different letters signify that the proportions are significantly different from one another.

Mentoring Outcomes	Mentor roles					
	Research	Coach	Connector	Sponsor	Peer	Identity
Publications	0.82^{a}	0.51^{c}	0.66^{b}	0.66^{b}	0.58^{bc}	0.41^{c}
Grants	0.74^{a}	0.52^{b}	0.57^{b}	0.61^{b}	0.47^{bc}	0.29^{c}
Skill	0.59^{a}	0.38^{b}	0.32^{b}	0.37^{b}	0.35^{b}	0.22^{b}
Guidance	0.74^{bc}	0.82^{a}	0.62^{c}	0.76^{abc}	0.81^{ab}	0.76^{abc}
Connections	0.62^{b}	0.51^{c}	0.82^{a}	0.73^{ab}	0.34^{c}	0.36^{c}
Positions	0.32^{b}	0.28^{b}	0.23^{bc}	0.44^{a}	0.14^{c}	0.16^{bc}
Visibility	0.46^{b}	0.31^{c}	0.49^{b}	0.69^{a}	0.20^{c}	0.21^{c}
Confidence	0.52^{bc}	0.62^{a}	0.39^{c}	0.52^{abc}	0.60^{ab}	0.62^{abc}
Growth	0.40^{b}	0.52^{a}	0.25^{c}	0.38^{abc}	0.51^{a}	0.48^{ab}
Work life	0.21^{bc}	0.22^{bc}	0.17^{bc}	0.15^{c}	0.40^{a}	0.34^{ab}
